# Dual-Branch Superpixel and Class-Center Attention Network for Efficient Semantic Segmentation

**DOI:** 10.3390/s25247637

**Published:** 2025-12-16

**Authors:** Yunting Zhang, Hongbin Yu, Haonan Wang, Mengru Zhou, Tao Zhang, Yeh-Cheng Chen

**Affiliations:** 1 School of Design Media, WuXi Vocational Institute of Commerce, Wuxi 214166, China; zhangyunting0630@163.com; 2 School of Artificial Intelligence and Computer Science, Jiangnan University, Wuxi 214126, China; 1193220403@stu.jiangnan.edu.cn (H.W.); zhoumengru1634@163.com (M.Z.); 3 School of Electronics and Information Engineering, Central South University, Changsha 410017, China; taozhang@jiangnan.edu.cn; 4 Department of Computer Science, University of California Davis, Davis, CA 95616, USA; ycch@ucdavis.edu

**Keywords:** image semantic segmentation, superpixel-guided attention, dual-branch network

## Abstract

With the advancement of deep learning, image semantic segmentation has achieved remarkable progress. However, the complexity and real-time requirements of practical applications pose greater challenges for segmentation algorithms. To address these, we propose a dual-branch network guided by attention mechanisms that tackles common limitations in existing methods, such as coarse edge segmentation, insufficient contextual understanding, and high computational overhead. Specifically, we introduce a superpixel sampling weighting module that models pixel dependencies based on different regional affiliations, thereby enhancing the network’s sensitivity to object boundaries while preserving local features. Furthermore, a class-center attention module is designed to extract class-centered features and facilitate category-aware modeling. This module reduces the computational overhead and redundancy of traditional self-attention mechanisms, thereby improving the network’s global feature representation. Additionally, learnable parameters are employed to adaptively fuse features from both branches, enabling the network to better focus on critical information. We validate our method on three benchmark datasets (PASCAL VOC 2012, Cityscapes, and ADE20K) by comparing it with mainstream models including FCN, DeepLabV3+, and DANet, with evaluation metrics of mIoU and PA. Our method delivers superior segmentation performance in these experiments. These results underscore the effectiveness of the proposed algorithm in balancing segmentation accuracy and model efficiency.

## 1. Introduction

Semantic segmentation of images is a prominent research topic in the field of computer vision, aiming to assign a class label to each individual pixel. It plays a critical role in various application domains, including autonomous driving, intelligent healthcare, and scene understanding [1,2,3,4,5,6].

Early semantic segmentation methods primarily relied on hand-crafted features such as color, texture, and edges. These approaches encompassed threshold-based methods [7,8], region-based methods [9,10,11], edge-based methods [12,13], and graph-based methods [14,15,16]. However, with the rapid advancement of computational hardware and deep learning techniques, these traditional methods have gradually been supplanted by approaches based on deep convolutional neural networks, leading to significant breakthroughs in semantic segmentation performance.

Long et al. [17] introduced Fully Convolutional Networks (FCNs), which transformed image-level classification into pixel-level prediction and enabled end-to-end training. Despite their impact, FCNs suffer from significant limitations: excessive downsampling leads to the loss of fine-grained spatial details, and the fixed receptive field of standard convolutions constrains the ability to model global contextual information. To mitigate these issues, subsequent FCN-based methods have incorporated techniques such as feature fusion, multi-scale processing, and dilated convolutions. For example, PSPNet [18] leveraged global pooling alongside pyramid modules to capture long-range contextual dependencies. The DeepLab series [19,20,21,22] adopted dilated convolutions with varying rates to extract multi-scale context.

While methods such as the DeepLab series effectively expand the receptive field through dilated convolutions, they remain primarily reliant on strategies rooted in local pixel-level modeling (rather than global pixel-wise modeling) to capture spatial information. This restricts their ability to effectively capture long-range dependencies and holistic contextual relationships among target objects.

In recent years, Attention mechanism-based semantic segmentation algorithms have attracted significant interest due to their exceptional ability to model global contextual information. Non-local Neural Networks [23] capture long-range dependencies by computing similarities between any two spatial positions, unrestricted to local neighborhoods. Building on this, PSANet [24] adaptively learns attention coefficients to establish contextual relationships by connecting each position on the feature map with others. DANet [25] employs a joint spatial and channel attention mechanism: the spatial attention component models correlations between any two pixels, while the channel attention captures relationships across feature channels, ultimately fusing these complementary features. However, the computational complexity of such self-attention mechanisms scales quadratically with the feature map size, posing challenges for pixel-level classification tasks that require maintaining high-resolution feature maps for accurate segmentation. To address this, CCNet [26] introduces a Criss-Cross Attention (CCA) module that leverages predicted sparse attention maps to capture pixel dependencies while reducing parameter overhead through parameter sharing. OCRNet [27] further proposes modeling correlations between pixels and category-level features to better capture object context.

Meanwhile, superpixel segmentation has been widely explored as a preprocessing step to improve efficiency and boundary precision. Classical methods such as SLIC [28], LSC [29], and SNIC [30] exploit spatial and color cues for clustering. More recent works, including SSN [31] and CNN-based superpixel generation [32], integrate deep features for end-to-end superpixel learning.

Based on the above research, we propose a dual-branch attention-guided semantic segmentation network (DAMNet). The network is designed to effectively capture both local and global contextual information while mitigating the computational overhead typically introduced by attention mechanisms. Specifically, DAMNet employs a two-branch architecture: one branch focuses on local feature extraction and the other targets global context modeling. By fusing the complementary strengths of both branches, the network alleviates the problem of pixel-wise independence caused by limited contextual awareness in conventional networks.

To enhance local feature representation, we design a superpixel sampling weighting module, which captures dependencies between pixels and their corresponding superpixels. This module leverages superpixel-based clustering to extract edge-aware and locally sensitive features. For global context modeling, we introduce a class-center attention module, which reframes pixel-to-pixel relationships as pixel-to-class-center relationships. By incorporating prior information to estimate class centers, this module reduces the redundancy and complexity of traditional self-attention while establishing long-range dependencies, improving the network’s ability to distinguish between semantic categories.

Finally, an adaptive feature fusion module is integrated to dynamically combine local and global features, enhancing the overall segmentation performance and generalization capability of the network. Through this architecture, DAMNet achieves a balance between accuracy and efficiency, addressing both fine-grained detail preservation and global contextual understanding in image semantic segmentation.

The main contributions of this paper are as follows:

1. We propose a dual-branch network architecture that effectively integrates local and global contextual information, thus enhancing overall segmentation performance.
2. We design a superpixel sampling weighting module that enriches local semantic features and enhances the network’s sensitivity to target boundaries.
3. We introduce a class-center attention module that performs category-aware modeling and extracts class-center features, enabling efficient global context capture.
4. The proposed method delivers superior performance on multiple benchmark datasets (PASCAL VOC 2012, Cityscapes, and ADE20K), which validates its effectiveness and robustness.

## 2. Method

**Figure 1:**
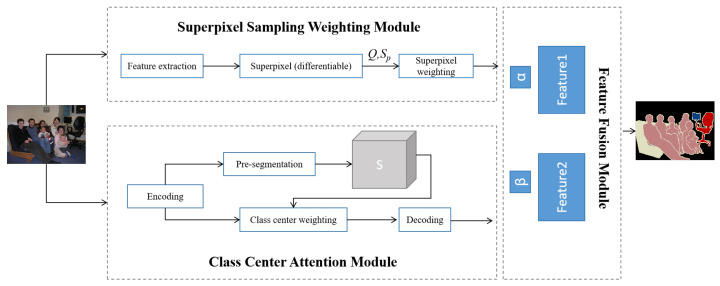
Diagram of the proposed algorithm.

The proposed network framework is illustrated in Figure 1 and consists of two branches: the superpixel sampling weighting module and the class-center attention module. In image semantic segmentation, global information typically corresponds to high-level semantic features, whereas local information is represented by the network’s shallow features. By effectively integrating these complementary feature types, the network enhances its understanding and representation of the target, thereby improving segmentation accuracy.

First, we employ a local superpixel sampling weighting strategy to enhance local feature representation. This method improves the model’s sensitivity to object boundaries while effectively capturing local details by modeling pixels with soft membership relationships and generating weighted features that preserve boundary information in a pixelated manner. Second, we introduce the concept of class centers to model global features. This approach transforms the traditional self-attention mechanism from modeling pixel-to-pixel interactions into modeling relationships between pixels and class centers, thereby reducing computational complexity and information redundancy. To capture global context, we update the pre-segmentation results by representing the similarity between pixels and class centers and then concatenate this information with the original feature map to enrich the feature representation. Finally, an adaptive feature fusion module is designed to effectively integrate local and global features, enhancing both segmentation accuracy and the network’s generalization capability.

### 2.1. Superpixel Sampling Weighting Module

The superpixel sampling weighting module is designed to capture local features of the image, providing fine-grained information for subsequent segmentation tasks. By leveraging the inherent advantages of superpixel segmentation in preserving object boundaries, this module incorporates superpixel-based local soft clustering into the deep network. This integration enhances the network’s focus on boundary information while effectively extracting local contextual features.

**Figure 2:**
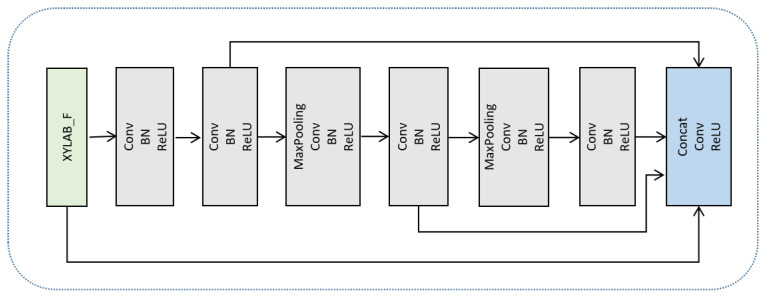
Feature extraction network.

The process begins with feature extraction from the input image, capturing essential low-level information such as edges and textures, which serve as input to the superpixel sampling stage. As illustrated in Figure 2, the feature extraction network is composed of a series of convolutional layers, batch normalization layers (BN), and ReLU activation functions. To increase the receptive field, max-pooling operations are applied after the third and fifth down-sampling stages. Furthermore, in order to integrate multi-scale information, low-level and high-level feature maps are concatenated to form enriched pixel-level features that support a more accurate superpixel-based representation.

Furthermore, to better utilize the relationship between pixels and superpixels, the hard association is transformed into a soft association, replacing nearest-neighbor constraints with weight computation. This approach provides a more flexible way to capture the mapping *Q* between pixels and superpixel features $S_{p}$. Specifically, for the *t*-th iteration, the mapping relationship between pixel *p* and superpixel *i* is given by (1). Additionally, during the iteration process, distance calculation is constrained to the nine superpixels surrounding each pixel. The corresponding *Q* becomes a sparse matrix, improving the computational efficiency of the algorithm while enhancing the capture of local pixel correlations.

(1) $$Q_{pi}^{t}=e^{-D(I_{p},S_{i}^{t-1})}=e^{-∥I_{p},S_{i}^{t-1}{∥}_{2}}$$

Finally, *Q* is normalized row-wise and column-wise to obtain $\tilde{Q}$ and $\hat{Q}$. The normalized results are then used to weight the superpixel features $S_{p}$ separately, followed by feature fusion in (2). The row-normalized $\tilde{Q}$ standardizes the pixel-to-superpixel membership relationships, representing the relationships between each pixel and superpixel. On the other hand, the column-normalized matrix $\hat{Q}$ standardizes the superpixel-to-pixel membership relationships, indicating the relationships between each superpixel and different pixels. This weighted fusion approach not only balances the relationships between pixels and superpixels but also addresses the issue of feature neglect caused by overly sparse matrices, enriching local feature information.

(2) $$Fus=\alpha \cdot \left(\tilde{Q}⊗S_{p}\right)+\left(1-\alpha \right)\cdot \left(\hat{Q}⊗S_{p}\right)$$

### 2.2. Class Center Attention Module

Self-attention mechanisms are effective in capturing long-range dependencies between pixels, enabling the model to develop a more comprehensive understanding of image content and thereby improving semantic segmentation accuracy. However, their high computational complexity introduces a significant trade-off between accuracy and inference speed when applied to convolutional neural networks.

**Figure 3:**
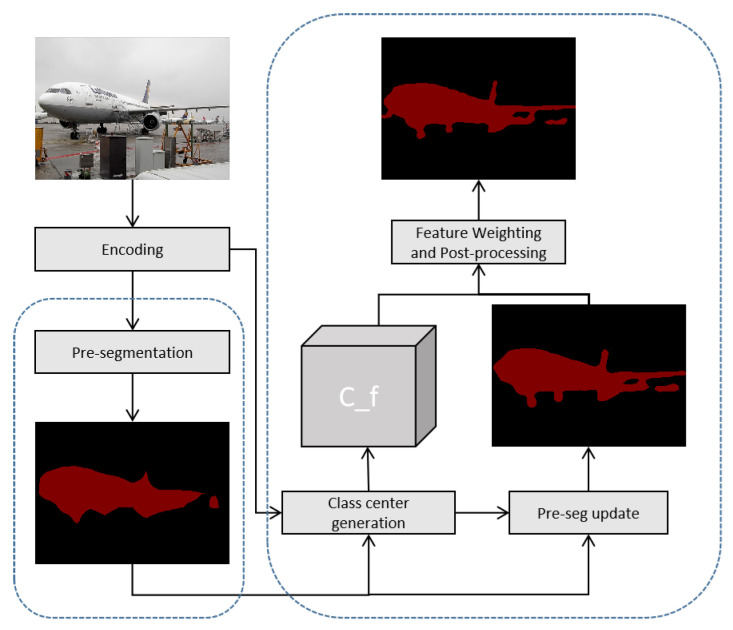
Network structure of class-center attention module.

To address this challenge, we propose a Class-Center Attention Module, illustrated in Figure 3, specifically designed to reduce computational overhead while preserving the benefits of global context modeling. This module utilizes pre-segmentation results to generate class center features, which serve as category-level representations. By transforming pixel-to-pixel associations into relationships between pixels and class centers, the module shifts the modeling perspective from the spatial domain to the semantic category domain. This transformation significantly reduces redundancy in the attention matrix and improves computational efficiency, while still enabling effective global feature extraction.

Firstly, feature information *F* is extracted using an encoding network and pre-segmentation results *S* are obtained based on a fully convolutional network, serving as prior information for subsequent operations. To address the issue of detail loss during the downsampling process, we make improvements based on ResNet50, consisting of five stages. The settings for stages 1 to 3 are consistent with the ResNet50 network, while in stages 4 and 5, the stride is set to 1, preserving the resolution of the feature maps. Additionally, dilated convolutions are introduced to replace ordinary convolutions, increasing the receptive field without adding extra parameters. The dilation factors in these two stages are set to {1, 2} and {2, 4}, progressively expanding the receptive field to capture more feature information.

Secondly, to reduce computational costs, the feature map *F* is dimensionally reduced using a 1 × 1 convolution, reducing the number of channels to $C^{\prime }$. Simultaneously, *F* and Seg are reshaped into $F\in R^{C^{\prime }\times N}$ and $Seg\in R^{N_{c}\times N}$, where N=H×W, *H* denotes height, and *W* denotes width. The pre-segmentation results *S* serve as the similarity between pixels and classes. Matrix multiplication is then performed on the transposes of *F* and Seg, followed by normalization, yielding the global class center features $F_{c}\in R^{C^{\prime }\times N_{c}}$. The calculation formula is as follows:

(3) $$F_{c}^{i}=\frac{\sum_{j=0}^{N}Sim^{i,j}\cdot F_{j}}{\sum_{j=0}^{N}Sim^{i,j}}$$

where $Sim^{i,j}$ represents the probability that pixel *j* belongs to class *i*.

**Figure 4:**
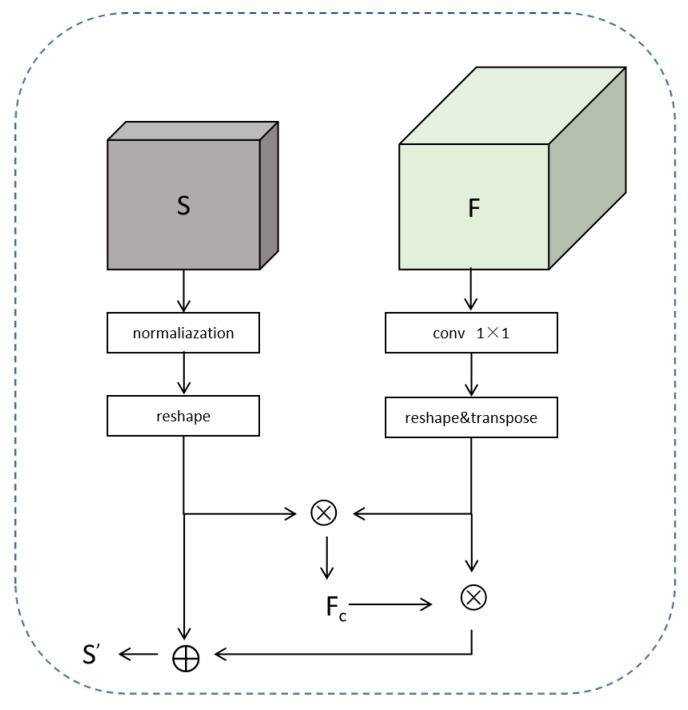
Flowchart of the Class Center Generation and Pre-segmentation Update Process.

Finally, the pre-segmentation results are updated using class center features (depicted in Figure 4), as shown in (4).

(4) $$Seg^{\prime }=\beta \cdot Seg+\left(1-\beta \right)\cdot \left(F⊗\hat{F_{c}}\right)$$

As evident from the formulation, the updated segmentation results rely on two key components: the initial pre-segmentation outputs and the feature similarity between class center features and individual pixel features. When the pre-segmentation result indicates a high probability of a pixel belonging to a specific class, that pixel contributes more strongly to the corresponding class center, and the similarity between the pixel and the class center feature is higher. As a result, the updated segmentation is more likely to assign that pixel to the same class.

Conversely, when the class probabilities for a pixel are relatively uniform—indicating weak feature discrimination—the introduction of long-range contextual information via class center features helps enhance class-specific distinctiveness. This, in turn, improves the reliability and accuracy of pixel-level classification.

To reduce the dependency on coarse pre-segmentation results, we introduce class center attention feature maps, computed by weighting the class center features into the updated segmentation representation. These attention feature maps are then reshaped to a size of $C^{\prime }\times H\times W$ and passed through a 1×1 convolution to align with the dimensions of the original feature map *F*. Finally, a straightforward element-wise addition is performed to fuse the original feature map with the refined class center attention map, resulting in semantic features enriched with global contextual information.

It is worth emphasizing that the design of this update mechanism cleverly avoids rigid reliance on the quality of initial pre-segmentation results, thereby alleviating the potential “circular dependency” issue. In this design, the initial pre-segmentation result *S* only serves as a dynamic “class prior” rather than the sole decision-making basis. As shown in (4), the updated segmentation result ${Seg}^{\prime }$ is obtained through adaptive weighted fusion of the initial pre-segmentation Seg and a term based on feature similarity $F⊗{\hat{F}}_{c}$. This means that even in the early stages of training, when pre-segmentation results are relatively coarse, the similarity calculation between pixel features *F* and their corresponding class-center features ${\hat{F}}_{c}$ can still provide a strong correction signal. If the feature of a certain pixel is closer to the feature of another class center, this term will dominate the update process, thereby effectively correcting the initial misclassification. More importantly, this mechanism forms a feedback loop for positive optimization during the training process. As training progresses, the feature representation capability of the encoder continues to improve, and the quality of pre-segmentation results is enhanced accordingly. Higher-quality pre-segmentation generates more accurate class centers, and these more accurate class centers can provide more reliable global contextual information through the attention mechanism, further optimizing feature representation. This iterative self-improvement process enables the module to converge stably and gradually improve the accuracy and robustness of segmentation.

### 2.3. Feature Fusion Module

The local information extracted by the Superpixel Sampling Weighting Module and the global information obtained from the Class-Center Attention Module are both complementary and partially overlapping, each contributing differently to the semantic segmentation task. To effectively integrate these two sources of information, we introduce an adaptive feature fusion mechanism, which enables the network to more comprehensively understand the semantic content of the image.

This mechanism incorporates learnable parameters to dynamically adjust the relative importance of local and global feature maps. By allowing the network to autonomously learn optimal fusion weights during training, the model enhances its feature representation capabilities, resulting in improved segmentation accuracy and robustness. The fusion is computed as follows:

(5) $$F=\lambda \cdot F_{s}+\left(1-\lambda \right)\cdot F_{a}$$

where $F_{s}$ is the feature map obtained from the superpixel sampling weighted module, $F_{a}$ is the feature map obtained from the class center attention module, and λ represents the learnable parameters.

### 2.4. Training Details

In model training, we utilized the stochastic gradient descent optimizer with a weight decay set to 0.0005 and the batch size uniformly set to 4. For the learning rate, we employed the “poly” adjustment strategy, represented by the formula

(6) $$lr=init\_lr\times {\left(1-\frac{epoch}{max_{e}poch}\right)}^{power}$$

where, init_lr denotes the initial learning rate, set to 0.0001 for PASCAL VOC 2012 and 0.01 for CityScapes and ADE20k. The max_epoch indicates the maximum number of iterations, set to 200, and the power was set to 0.9.

To prevent overfitting, we applied common data augmentation strategies to process images, including random scaling, random horizontal flipping, and random cropping.

### 2.5. Evaluation Metrics

In the field of image segmentation, several metrics are employed to evaluate model performance. This paper adopts a set of widely used metrics, including Pixel Accuracy (PA), Mean Intersection over Union (mIoU), Boundary Recall (BR), and Achievable Segmentation Accuracy (ASA). These metrics comprehensively assess the segmentation effectiveness of the algorithm.

Pixel Accuracy (PA) represents the ratio of correctly segmented pixels to the total number of pixels. Performance is directly proportional to the PA value; a higher PA value indicates better overall performance. The formula for PA is given by

(7) $$PA=\frac{\sum_{i=0}^{n}\;p_{ii}}{\sum_{i=0}^{n}\;\sum_{j=0}^{n}\;p_{ij}}$$

where *n* is the total number of semantic categories excluding the background, *i* represents the ground truth class, *j* represents the predicted class, and $p_{ij}$ denotes the number of pixels of class *i* that are predicted as class *j*.

Mean Intersection over Union (mIoU) measures the degree of overlap between the segmentation result and the ground truth. It is defined as the average of the ratio of intersection to union between the predicted segmentation and the ground truth for each class. A higher mIoU value signifies that the algorithm’s segmentation result aligns more closely with the ground truth image. The formula for mIoU is given following:

(8) $$MIOU=\frac{1}{n+1}\sum_{i=0}^{n}\frac{p_{ii}}{\sum_{j=0}^{n}\;p_{ij}+\sum_{j=0}^{n}\;p_{ji}-p_{ii}}$$

In the aforementioned formula, n+1 represents the total number of semantic categories including the background. The term $p_{ii}$ represents the true positives (pixels of class *i* correctly predicted as class *i*). The denominator $\sum_{j=0}^{n}\;p_{ij}+\sum_{j=0}^{n}\;p_{ji}-p_{ii}$ calculates the union of the predicted and ground truth pixels for class *i*. This can also be expressed for each class using True Positives (TP), False Positives (FP), and False Negatives (FN), with mIoU being the mean of this value across all classes:

(9) $$MIOU=\frac{1}{n+1}\sum_{i=0}^{n}\frac{TP}{TP+FP+FN}$$

Unlike PA in (7), which treats the contribution of each pixel equally, mIoU provides a distinct evaluation of performance for each class, offering a more comprehensive and objective reflection of the algorithm’s capabilities.

Boundary Recall (BR) and Achievable Segmentation Accuracy (ASA) are primarily used to evaluate the performance of superpixel segmentation algorithms. BR is used to measure the effectiveness of boundary segmentation, representing the proportion of ground truth boundaries that are correctly detected (or “recalled”) by the predicted segmentation boundaries within a given tolerance.

(10) $$Rec\left(G,S\right)=\frac{TP(G,S)}{TP(G,S)+FN(G,S)}$$

where *G* represents the set of ground truth superpixel boundaries and *S* is the set of predicted superpixel boundaries. TP(G,S) denotes the number of ground truth boundary pixels in *G* that are correctly identified by a predicted boundary pixel in *S* within a specified neighborhood. FN(G,S) denotes the number of ground truth boundary pixels in *G* that are not detected by any predicted boundary pixel in *S* within that neighborhood.

Achievable Segmentation Accuracy (ASA) evaluates the accuracy of superpixel segmentation from a pixel-level perspective by measuring the similarity between the predicted superpixels and the ground truth superpixels. It calculates the upper-bound accuracy achievable by labeling each predicted superpixel with the ground truth label that has the maximum overlap.

(11) $$ASA\left(G,S\right)=\frac{1}{N}\sum_{S_{j}}\max_{G_{i}}\left\{\right|S_{j}\cap G_{i}\left|\right\}$$

where *S* is the set of predicted superpixel blocks ($S_{j}$ being the *j*-th superpixel), *G* is the set of ground truth superpixels ($G_{i}$ being the *i*-th ground truth superpixel), and *N* is the total number of pixels in the image. The term $|S_{j}\cap G_{i}|$ represents the count of pixels in the intersection of the predicted superpixel $S_{j}$ and the ground truth superpixel $G_{i}$.

## 3. Experiments

Extensive experiments were conducted on several benchmark datasets widely used in image semantic segmentation, including PASCAL VOC 2012, Cityscapes, and ADE20K, to evaluate the effectiveness of the proposed method. In this section, we first introduce the datasets, evaluation metrics, and implementation details. Next, we conduct a series of ablation studies to verify the individual contributions of each module within the network. Finally, we compare our method with state-of-the-art segmentation models to demonstrate its competitive performance and overall feasibility.

### 3.1. Dataset

#### 3.1.1. PASCAL VOC 2012

PASCAL VOC 2012 is a widely used dataset for image semantic segmentation. This dataset comprises 20 distinct object categories, such as people, cars, dogs, birds, and chairs. We conducted experiments using the standard edition, which includes 11,540 images. Among them, 9963 images are used for training, 1449 for validation, and 1028 for testing.

#### 3.1.2. CityScapes

CityScapes is a high-resolution image dataset focused on urban scenes, with each image having a resolution of 2048×1024 pixels. The dataset consists of over 5000 images, with 2975 images used for model training, 500 for validation, and 1525 for testing. Each pixel in the images is annotated with a different semantic category, such as people, cars, roads, buildings, trees, etc. In this paper, we trained and evaluated the model using dense pixel annotations for 19 semantic classes.

#### 3.1.3. ADE20k

ADE20k is an extensive high-resolution dataset used in many computer vision applications. For this study, we utilized the processed dataset provided by ADEchallenge 2016, which includes 22,210 images and covers 150 classes (excluding the background). The training set consists of 20,210 images with completely annotated labels, while the validation set contains 2000 images with complete annotations.

### 3.2. Experimental Configuration

The proposed method is implemented on a machine equipped with an NVIDIA GeForce RTX 4090 GPU using the PyTorch framework. To ensure fair comparisons, all benchmarking experiments were conducted on this machine.

### 3.3. Ablation Experiments

To confirm the efficacy of the different modules suggested in the network model, a number of ablation experiments were carried out on the CityScapes dataset in this section.

#### 3.3.1. Different Weighting Methods

In the Superpixel Sampling Weighting Module, to investigate the impact of different weighting methods on segmentation performance, experiments were conducted based on the SSN [31] superpixel segmentation algorithm, and the experimental results are shown in Table 1.

The P2SPA (Pixel-to-Superpixel Attention) weighting method involves row normalization of the pixel-to-superpixel mapping matrix, followed by an attention operation using superpixel features. This balances the influence of each pixel across multiple associated superpixels, ensuring more equitable feature contribution. In contrast, the SP2PA (Superpixel-to-Pixel Attention) weighting method applies column normalization to the same mapping matrix and computes attention between superpixels and pixels. This approach enhances the representation of how each superpixel relates to various pixels, thereby balancing the contribution of superpixels to different pixel locations.

As shown in the table, incorporating either P2SPA or SP2PA into the baseline network consistently improves segmentation performance, confirming the effectiveness of integrating pixel–superpixel affinity relationships in semantic segmentation tasks. Among the final three experimental configurations, the combined use of both P2SPA and SP2PA achieves the best performance. This is attributed to the complementary nature of the two mechanisms: while P2SPA captures the associations between individual pixels and superpixels, SP2PA effectively models the relationships between superpixels and their neighboring pixel regions. Furthermore, the combination of row and column normalization mitigates feature sparsity issues arising from mapping relations, resulting in richer and more informative local features.

Based on these observations, we adopt the P2SPA+SP2PA fusion strategy in the Superpixel Sampling Weighting Module for optimal performance.

#### 3.3.2. Different Attention Mechanisms

To validate the effectiveness of the Class Center Attention Module, we compared the segmentation performance of different attention mechanisms in the baseline network, and the experimental results are shown in Table 2.

A comparison between the baseline network and other variants incorporating different attention mechanisms demonstrates the effectiveness of introducing attention mechanisms into semantic segmentation models. By enabling the capture of global contextual information, attention mechanisms significantly enhance segmentation accuracy. Among the networks employing various attention strategies, the model incorporating our Class-Center Attention Module achieves the best performance.

This improvement can be attributed to the module’s ability to transform traditional pixel-to-pixel relationships into pixel-to-class-center associations, which enables the network to establish long-range dependencies based on class-level global features. This transformation not only facilitates the acquisition of richer contextual information but also reduces redundancy within the pixel correlation matrix. As a result, it helps mitigate noise interference and improves the overall discriminative power of the segmentation network.

#### 3.3.3. Differential Feature Fusion Methods

To investigate the impact of different feature fusion methods on algorithm performance, we compared their segmentation performance on the CityScapes dataset, and the experimental results are shown in the Table 3.

In the “add” fusion method, the feature maps from the two branches are combined through element-wise addition. Specifically, the feature map generated by the Class-Center Attention Module is first upsampled via bilinear interpolation to match the original input resolution. It is then added to the corresponding feature map from the Superpixel Sampling Weighting Module at each spatial position.

In the “concat” fusion method, the two feature maps are concatenated along the channel dimension. Similarly, the output of the Class-Center Attention Module is upsampled to match the spatial resolution of the Superpixel Sampling Weighting Module. The two are then combined through channel-wise concatenation. A final 1×1 convolution is applied to adjust the number of channels to match the number of semantic categories in the dataset, producing the final segmentation output.

Experimental results demonstrate that the proposed adaptive weighted feature fusion method outperforms both the “add” and “concat” strategies, validating its effectiveness. Unlike fixed fusion operations, the adaptive method learns to assign dynamic weights to different branches, allowing the network to more effectively integrate complementary feature information. This approach enhances focus on informative features while suppressing irrelevant ones, ultimately improving segmentation accuracy and overall model robustness.

### 3.4. Comparative Experiments

To validate the effectiveness of the proposed method, we conducted comparative analyses with mainstream algorithms on the PASCAL VOC 2012, CityScapes, and ADE20k datasets.

#### 3.4.1. Comparative Experiments on the PASCAL VOC 2012 Dataset

Table 4 presents the segmentation performance of various algorithms on the PASCAL VOC 2012 dataset. A comparative analysis of the experimental results indicates that the proposed algorithm achieves superior segmentation accuracy, demonstrating its effectiveness in capturing and integrating both local and global contextual information. This capability significantly contributes to improved segmentation performance.

Although DSANet and JPANet exhibit advantages in terms of parameter efficiency and runtime speed, their segmentation accuracy falls noticeably short compared to the proposed method. This highlights a trade-off in these models between computational efficiency and segmentation quality.

In summary, the proposed algorithm successfully enhances segmentation accuracy while maintaining a relatively low parameter count. It addresses the inherent challenge of balancing segmentation precision and model efficiency, thereby offering a promising solution for practical semantic segmentation tasks.

**Figure 5:**
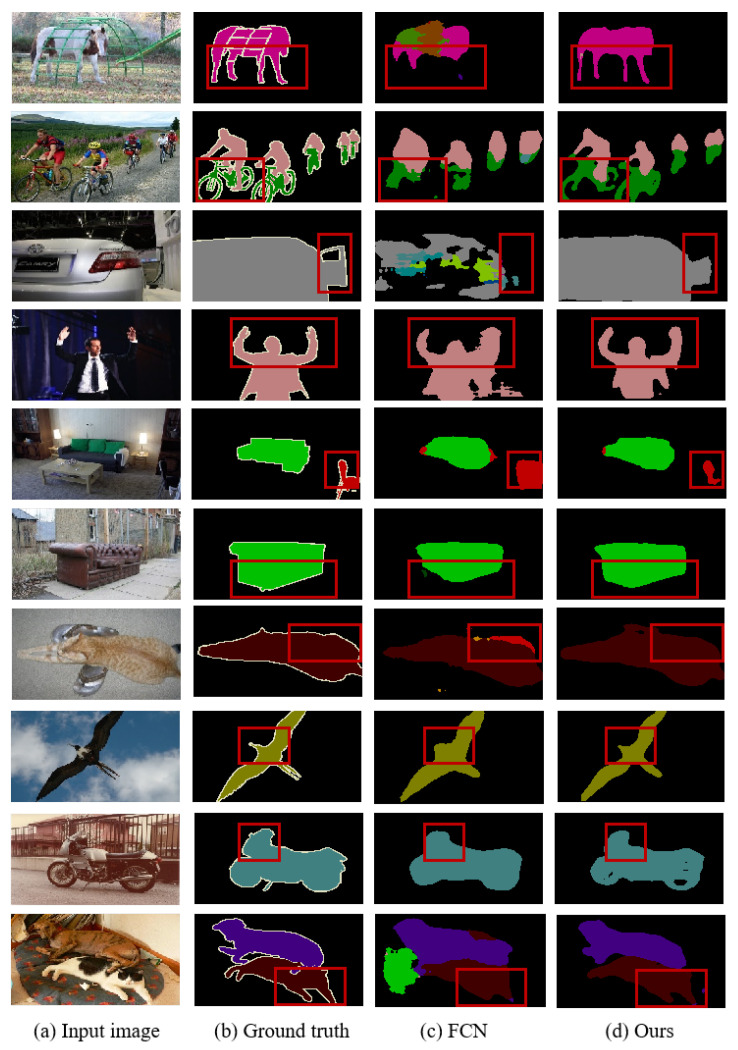
Visualization results on PASCAL VOC 2012 val set.

To visually validate the effectiveness of the proposed algorithm, Figure 5 presents the visual segmentation results on the PASCAL VOC 2012 dataset compared to baseline algorithms. Comparative analysis reveals that the proposed algorithm improves the segmentation results, particularly in capturing finer details at the edges. For instance, details such as horse legs, bicycle components, bird beaks, and motorcycle features exhibit increased refinement in segmentation results due to the algorithm’s ability to capture rich contextual information. Additionally, the algorithm enhances feature recognizability, reducing misclassifications, as seen in instances like the horseback, cars, cats, and carpets in the images.

#### 3.4.2. Comparative Experiments on the Cityscapes Dataset

**Figure 6:**
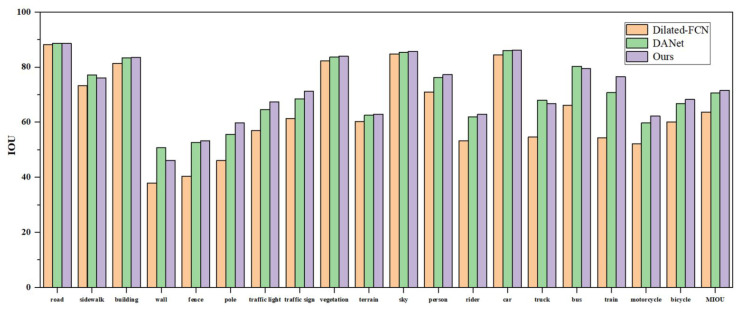
IoU scores for each class on the Cityscapes dataset.

Figure 6 presents a comparison of segmentation results across various categories for the baseline network, DANet, and the proposed algorithm.

The experimental results indicate that incorporating attention mechanisms significantly enhances segmentation performance. Both DANet and the proposed algorithm achieve higher IoU values across most categories compared to the baseline network, underscoring the importance of contextual information. While DANet utilizes self-attention mechanisms, our algorithm achieves superior segmentation performance, validating the effectiveness of integrating both local and global features.

A category-wise analysis reveals that for structurally simple and spatially concentrated classes—such as road, fence, vegetation, and sidewalk—both DANet and the proposed algorithm yield comparable segmentation results, demonstrating robust performance. However, for small-scale target objects like poles, traffic signs, pedestrians, and traffic signals, our method outperforms DANet, highlighting the effectiveness of the superpixel sampling weighting module in capturing fine-grained local information.

In summary, the proposed algorithm effectively combines local and global contextual cues, enabling the modeling of long-range dependencies while preserving edge detail, thereby further enhancing segmentation accuracy.

Table 5 presents a comparative evaluation between the proposed algorithm and several mainstream methods on the Cityscapes dataset. The results demonstrate that our method achieves the highest segmentation accuracy, confirming the effectiveness of incorporating contextual information through superpixels and class centers. Although our model has a higher parameter count than FCN, SegNet, DSANet, and JPANet, these lightweight networks exhibit relatively poorer performance and fail to maintain segmentation accuracy. Notably, compared to DANet—which also leverages attention mechanisms—our algorithm delivers superior segmentation accuracy with only half the number of parameters, striking a more effective balance between accuracy and model complexity.

**Figure 7:**
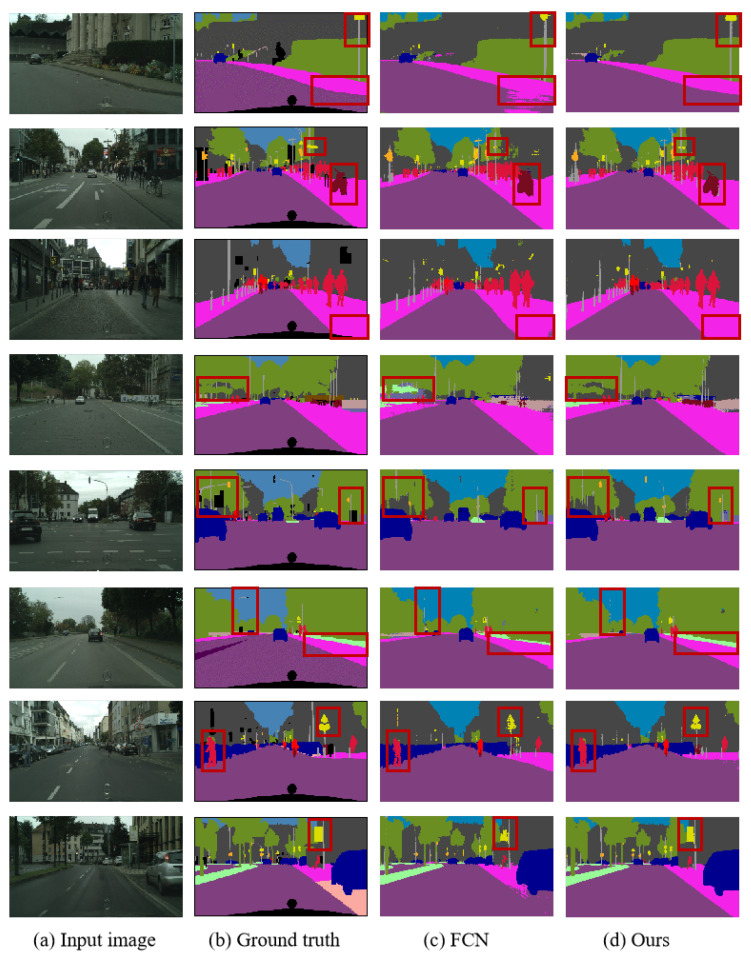
Visualization results on the Cityscapes val set.

The visualized semantic segmentation results on the CityScapes dataset are depicted in Figure 7. Through comparison, it can be observed that our algorithm improves the misclassification within object regions and optimizes the segmentation results for edge details. Notably, this enhancement is evident in areas such as roads, pedestrians, streetlights, and traffic signs. The experimental results demonstrate that the integration of global and local information in the network model can refine edge segmentation results, reduce misclassifications, and thereby enhance overall segmentation performance.

#### 3.4.3. The Comparative Experiment on the ADE20k Dataset

To further validate the robustness and generalization capability of our proposed algorithm, experiments were conducted on the ADE20K dataset. Table 6 presents a comparison of segmentation performance across various algorithms.

**Figure 8:**
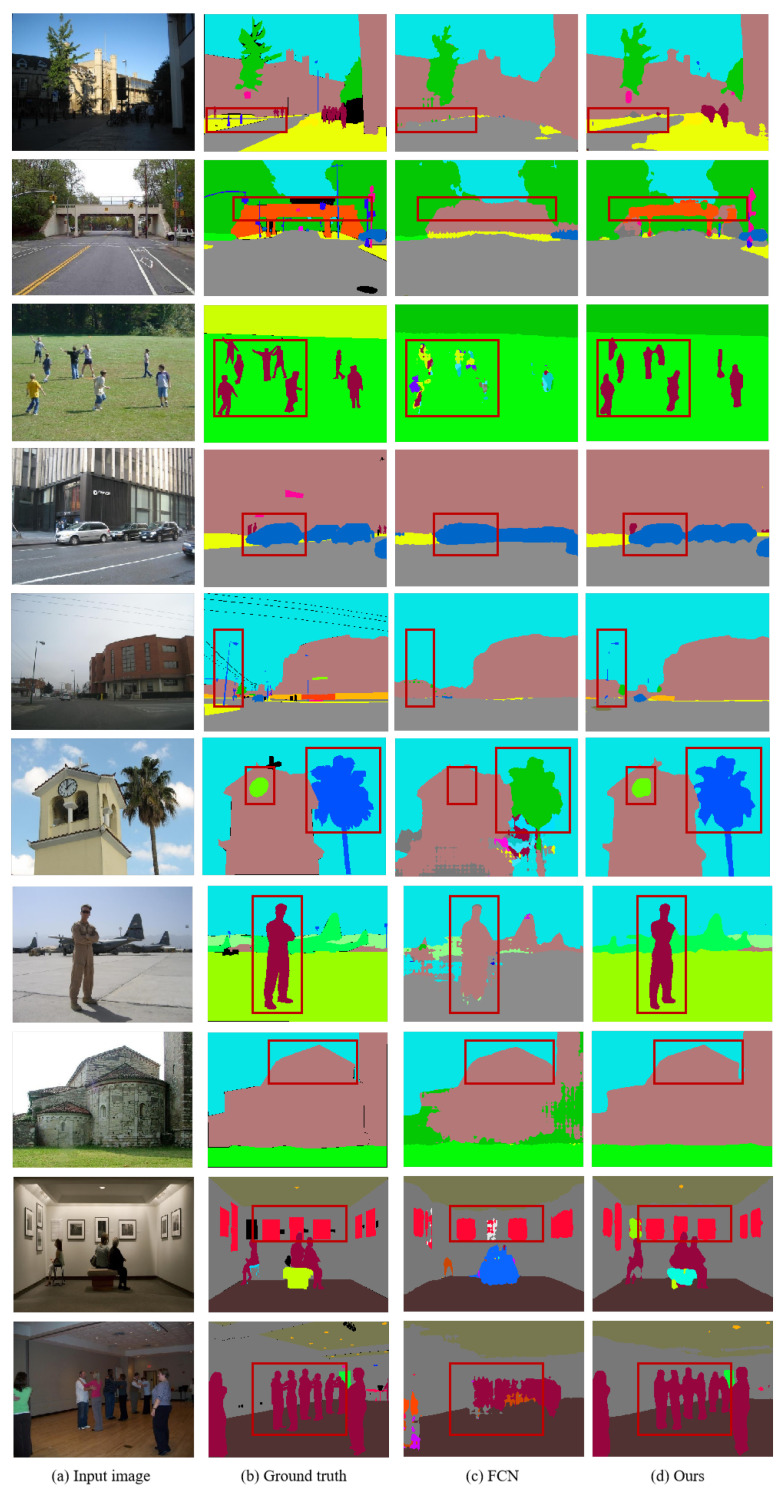
Visualization results on the ADE20k val set.

The experimental results demonstrate that our algorithm, through the Superpixel Weighting Module and Class-Center Attention Module, captures both local and global features of the image and fuses them, improving the network’s segmentation accuracy. Segmentation performance of the baseline network with our proposed algorithms is demonstrated in Figure 8. It can be noticed that the proposed algorithm can ameliorate misclassification issues, such as trees near the villa, balconies, and road surfaces. Furthermore, our method significantly improves the segmentation accuracy of small objects, such as traffic signs, lamp posts, building clocks, and pillows, with the help of local knowledge. To sum up, the outcomes of the experiments conducted on the ADE20k dataset provide additional confirmation of the efficiency and resilience of the suggested method within the domain of computer science.

#### 3.4.4. The Comparative Experiment on the Camvid Dataset

From Table 7, it can be observed that compared to the current state-of-the-art algorithms, our algorithm achieves optimal segmentation accuracy. Moreover, in comparison to several models with better parameter metrics, our algorithm significantly outperforms them in segmentation accuracy. Additionally, it is noteworthy that the DANet network, which performs well in segmentation on PASCAL VOC 2012, CityScapes, and ADE20k, does not stand out on this dataset. This further validates that our algorithm effectively balances segmentation accuracy and model complexity through local clustering and class center modeling.

**Figure 9:**
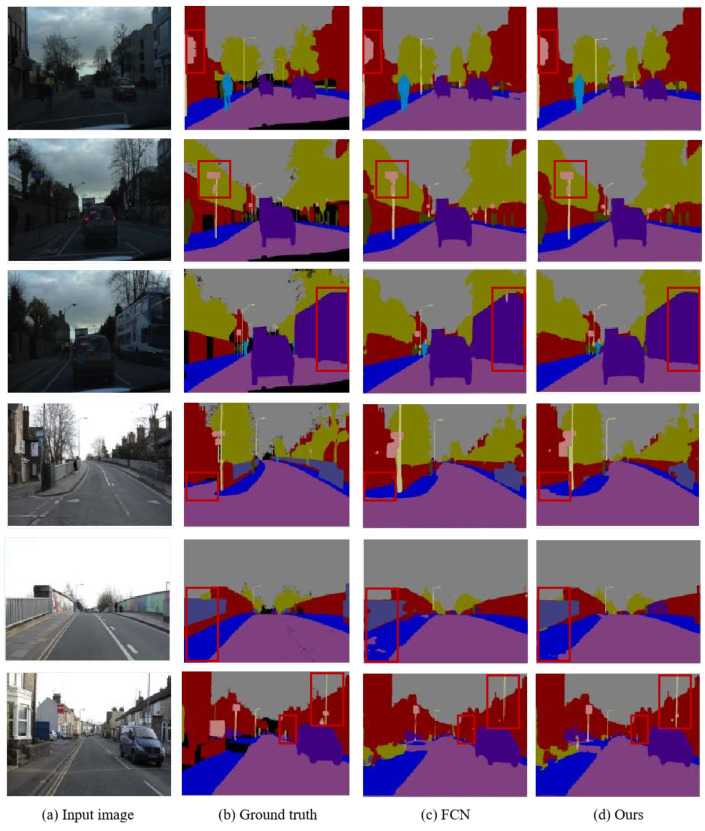
Visualization results on the CamVid validation set.

Figure 9 presents a comparison of the visual segmentation results between the baseline model and the proposed algorithm. It is evident that the proposed method improves segmentation accuracy and boundary regularity for small objects such as traffic signs and lamp posts. Compared to FCN, our approach incorporates a superpixel sampling weighting module to capture fine-grained local features while enhancing the network’s sensitivity to target boundaries, leading to smoother and more precise edge delineation. For larger objects like buildings and sidewalks, the improved algorithm leverages class-center modeling to incorporate rich contextual information, which significantly enhances feature discriminability, promotes spatial continuity in segmentation, and reduces instances of misclassification.

## 4. Conclusions

This paper addresses the challenges of missing contextual semantic information and high model complexity by proposing a dual-branch image semantic segmentation algorithm guided by attention mechanisms. The framework consists of three key components: a Superpixel Weighting Module, a Class-Center Attention Module, and an Adaptive Feature Fusion Module.

The Superpixel Weighting Module leverages the soft membership relationships between pixels and superpixels to capture local dependencies, thereby enriching local semantic features and enhancing the network’s sensitivity to object boundaries. The Class-Center Attention Module utilizes prior information to extract class center features and establishes long-range dependencies by modeling relationships between pixels and these class centers. This design effectively reduces computational complexity and information redundancy, enabling efficient extraction of global semantic features with rich contextual information. The Adaptive Feature Fusion Module dynamically adjusts the contributions of local and global features through learnable parameters, improving the network’s focus on salient information while suppressing irrelevant interference.

Extensive experiments conducted on the PASCAL VOC 2012, Cityscapes, and ADE20K datasets demonstrate that the proposed algorithm significantly enhances the network’s ability to capture contextual information, reduces misclassification, refines edge segmentation, and ultimately improves segmentation accuracy. Moreover, compared to other attention-based semantic segmentation networks, the proposed method reduces model complexity, thereby improving segmentation efficiency.

## Figures and Tables

**Table 1:** Performance Comparison of Different Weighting Methods.

Algorithm	BR (%)	ASA (%)
baseline	93.12	95.63
baseline+P2SPA	93.23	95.73
baseline+SP2PA	93.16	95.88
baseline+Ours	**94.05**	**96.26**

**Table 2:** Performance Comparison of Different Attention Mechanisms.

Algorithm	PA (%)	MIOU (%)
baseline	90.75	53.42
baseline+CAM [4]	91.23	57.34
baseline+BAM [33]	91.26	57.16
baseline+CBAM [34]	92.35	63.23
baseline+CA [35]	92.64	66.87
baseline+SA [36]	92.73	67.03
baseline+Ours	**92.85**	**68.74**

**Table 3:** Performance Comparison of Different Feature Fusion Methods.

Methods	PA (%)	MIOU (%)
add	92.61	66.54
concat	93.18	67.32
Ours	**94.35**	**71.86**

**Table 4:** Performance Comparison of Different Algorithms on the PASCAL VOC 2012 Dataset.

Algorithm	MIOU (%)	Times (ms)	Params (M)
FCN [17]	57.42	-	30.04
SegNet [37]	58.97	-	28.62
PSPNet [18]	62.83	-	65.71
DeepLabV3+ [22]	66.52	-	45.69
PSANet [24]	67.34	-	61.13
DANet [25]	71.75	38.47	95.53
DSANet [38]	66.86	**6.31**	**3.47**
JPANet [39]	66.47	6.82	3.49
MagNet [40]	68.56	29.3	6.37
Ours	**73.69**	30.35	40.28

**Table 5:** Performance Evaluation of Various Algorithms.

Algorithm	PA (%)	MIOU (%)	Params (M)
FCN [17]	90.32	49.63	30.04
SegNet [37]	89.76	52.39	28.62
PSPNet [18]	91.26	58.46	65.71
DeepLabV3+ [22]	92.15	62.04	45.69
PSANet [24]	91.57	60.05	61.13
DANet [25]	92.87	69.25	95.53
DSANet [38]	92.54	66.46	**3.47**
JPANet [39]	91.83	66.02	3.49
MagNet [40]	92.53	68.20	6.37
Ours	**93.66**	**71.86**	40.28

**Table 6:** Performance Comparison of Different Algorithms on ADE20k Dataset.

Algorithm	PA (%)	MIOU (%)	Params (M)
FCN [17]	70.56	26.69	30.04
SegNet [37]	71.30	29.15	28.62
PSPNet [18]	72.64	28.57	65.71
DeepLabV3+ [22]	75.27	30.73	45.69
PSANet [24]	76.29	29.48	61.13
DANet [25]	81.38	31.63	95.53
DSANet [38]	77.91	30.07	**3.47**
JPANet [39]	76.95	30.62	3.49
Ours	**82.19**	**32.66**	40.28

**Table 7:** Performance Comparison of Different Algorithms.

Algorithm	PA (%)	MIOU (%)	Params (M)
FCN [17]	89.56	50.32	30.04
SegNet [37]	90.83	51.67	28.62
ENet [41]	88.92	49.25	35.71
DeepLabV3+ [22]	93.16	69.78	45.69
BiSeNet [42]	91.05	63.54	49.02
DANet [25]	92.45	67.26	95.53
DSANet [38]	91.81	65.94	**3.47**
JPANet [39]	91.18	63.27	3.49
Ours	**93.74**	**70.66**	40.28

## Data Availability

The original data presented in the study are openly available in: CamVid Dataset at https://www.kaggle.com/datasets/carlolepelaars/camvid (accessed on 30 July 2025); ADE20k Dataset at https://ade20k.csail.mit.edu (accessed on 30 July 2025); Cityscapes Dataset at https://www.cityscapes-dataset.com (accessed on 30 July 2025); PASCAL VOC 2012 Dataset at https://www.kaggle.com/datasets/gopalbhattrai/pascal-voc-2012-dataset (accessed on 30 July 2025).
